# Individualised Homeopathic Therapy in ANCA Negative Rapidly Progressive Necrotising Crescentic Glomerulonephritis with Severe Renal Insufficiency – A Case Report

**DOI:** 10.25122/jml-2019-0001

**Published:** 2019

**Authors:** Seema Mahesh, Latika Jaggi, Atul Jaggi, Dionysios Tsintzas, George Vithoulkas

**Affiliations:** 1. Centre for Classical Homeopathy, Bangalore, India; 2. H3 Centre for Classical Homeopathy, Nashik, India; 3. Orthopaedic Department, General Hospital of Agrinio, Greece; 4. International Academy of Classical Homeopathy, University of the Aegean, Greece

**Keywords:** ANCA-negative, Dialysis free, Chronic renal insufficiency, Glomerulonephritis, Homeopathy, RPGN: Rapidly Progressing Glomerulonephritis, GFR: Glomerular Filtration Rate, ANCA: Anti-Neutrophil Cytoplasmic Antibody, AAV: ANCA-Associated Vasculitis, BVAS: Birmingham Vasculitis Score, CGN: Crescentic Glomerulonephritis, pANCA: Peripheral ANCA, cANCA: Cytoplasmic ANCA

## Abstract

Anti-Neutrophil Cytoplasmic Antibody (ANCA)-negative Rapidly Progressive Glomerulonephritis (RPGN) is a severe form of autoimmune renal injury with a bleak prognosis.

A 60-year-old Indian woman was treated with classical homeopathy for ANCA-negative RPGN, and after one year of treatment, serum creatinine and other parameters indicating renal injury dropped steadily despite the withdrawal of immunosuppressive drugs; renal dialysis, which was conducted twice a week initially, was made rarer and stopped after one year.

Classical homeopathy may be considered a potential therapeutic modality in severe pathologies. Controlled studies are required to establish further the extent to which classical homeopathy may relieve patients from procedures such as dialysis that cause considerable physical and economic discomfort.

## Introduction

Crescentic Glomerulonephritis (CGN) causes loss of renal function rapidly through cellular proliferation within Bowman’s space and formation of crescents. CGN is further differentiated on the presence of glomerular deposition of immune complexes seen on immunofluorescence. However, the majority of the CGN is pauci-immune, exhibiting no such deposits. These are termed ANCA-associated vasculitis (AAV) as these exhibit renal small-vessel vasculitis [1].

ANCA-negative RPGN is a diagnostic category of AAV that has not been studied as exhaustively as the ANCA-positive cases. Only 10-15% of the pauci-immune RPGN cases are ANCA-negative [2], and it affects younger people with very little extrarenal involvement when compared to the ANCA-positive. The renal damage is much higher and the prognosis poorer. Though mortality is not different from the positive cases, the dependency on dialysis is higher, and the renal improvement with immunosuppression or plasmapheresis is minimal [3, 4].

In these cases, though histologically renal damage may be extensive, the renal outcome after treatment is more significantly related to the serum creatinine at first consultation, and those with severe renal disease remained dialysis dependent [1–10]. Studies have shown renal outcome to be poor in ANCA-negative cases with very less probability for becoming dialysis-free [4, 11]. The negative prognostic factors for the renal outcome for CGN in general are: Glomerular Filtration Rate (GFR) <15 mL/min, advancing age, higher Birmingham Vasculitis Activity Score (BVAS), low hemoglobin and higher WBC count [1].

Immunosuppressive drugs such as cyclophosphamide prescribed in these cases have their risks associated and may be the cause for the increased mortality in older patients with ANCA-negative RPGN, due to cardiovascular diseases and infectious complications associated with immunosuppression [7].

The following case was diagnosed with ANCA-negative RPGN with severe renal insufficiency and underwent conventional treatment for 4 months with immunosuppressive drugs, dialysis, and plasmapheresis. The patient was receiving dialysis twice a week at the time of the homeopathic consultation, with high serum creatinine and low hemoglobin. The evolution of the case under homeopathic treatment is presented here. To the best of our knowledge, this is the first case report of this diagnosis under homeopathic treatment.

## Case Presentation

A 60-year-old Indian woman was diagnosed with rapidly progressing necrotizing crescentic glomerulonephritis with severe renal insufficiency in March 2015 (Table 1). She presented with a serum creatinine of 4.8 mg/dl, hematuria and albuminuria (GFR 9 mL/min/1.73 m^2^).On immunofluorescence testing, she was weakly positive for Anti Nuclear Antibodies but negative for both pANCA and cANCA. The lactate dehydrogenase, depicting the extent of tissue damage, was very high (404 IU/L; Normal: 103 - 227 IU/L). BVAS was estimated to be 14.

**Table 1:** Laboratory findings at the moment of diagnosis (09/03/2015) and medication before homeopathic therapy

Test	Patient value	Normal range	List of medications the patient was on, with content
**RBC count**	2.78 X 10^6^/cumm	3.5 – 5.5 X10^6^/cmm	Auxisoda (Sodium bi carbonate)
**Hemoglobin**	8.7 g%	11 to 16 g%	Calcigard (Nifedipine)
**Blood urea**	134.7 mg/dl	15 – 45 mg/dl	Alprax (Alprazolam)
**Serum creatinine**	4.8 mg/dl	0.6 – 1.4 mg/dl	Aciloc (rantidine)
**Estimated GFR**	9 mL/min/1.73 m^2^	> 60 mL/min/1.73 m^2^	Ondem (Ondansteron)
**Estimated BVAS**	14	NA	Frusenex (Furosemide)
**Serum albumin**	3.0 g/dl	3.2 – 4.6 g/dl	Metoz (Metolazone)
**Serum globulin**	2.1 g/dl	2.3 – 3.5 g/dl	Aldactone (Spironolactone)
**Total serum proteins**	5.1 g/dl	6 – 7.8 g/dl	Omnacortil (Prednisolone)
**Lactose dehydrogenase**	404.4 IU/L	103 - 227 IU/L	Endoxan (Cyclophosphamide)
**Reticulocyte count**	4%	0.2 – 2 %	Dargen (Darbepoetin)
**Antinuclear Antibody**	weakly +ve	-ve	Vozuca (Voglibose)
**pANCA**	-ve	NA	Linid (Linezolid)
**cANCA**	-ve	NA	Cardivas (Carvedilol)
**Urine albumin**	2+	nil	Ciplox (Ciprofloxacin)
**Urine RBC**	35 - 40 hpf	nil	
**Urine Protein to Creatinine Ratio**	2.64	<0.5	
**Abdominal and pelvic ultrasound scans**	Bilateral medical renal disease (Grade II)	NA	

She underwent conventional medical treatment until July. Initially, she received glucocorticoid and cyclophosphamide (immunosuppressive drugs) which did not control the serum creatinine. She then had to undergo plasmapheresis (5 sessions) and dialysis once a week. Despite this, the serum creatinine rose again, and the dialysis was increased to twice a week. However, there was no effective control of serum creatinine.

On 2/07/2015, with dialysis twice a week and immunosuppressive drugs, the serum creatinine was 5.2 mg/dl (normal is up to 1.4 mg/dl), GFR was 8 mL/min/1.73 m^2^, and hemoglobin was 8.7 g% (with a bone marrow-stimulating injection given periodically). The patient was already developing constitutional symptoms due to the immunosuppressive drugs, with weakness, loss of appetite, weight loss, pigmentation of skin and nails and shortness of breath. The BVAS (worsening) at this point worked out to be 15.

## Homeopathic intervention

The patient sought homeopathic treatment on 16/07/2015. The homeopathic case-taking involves an exhaustive recording of the patient’s past medical history, treatment history and also significant events that may have had a stressful effect along with the familial medical history. The intention is to arrive at the factors that have undermined the defense mechanism of the organism. In this case, the patient related that she started having problems 5 months earlier after severe stress from the illness of her mother. It began as edema, and she was diagnosed with ANCA RPGN.

In the past, she had developed a severe form of skin eruptions which were treated conventionally. She had undergone ozone therapy for arthritis of the knee a year and a half earlier. Family history revealed that her paternal grandfather and father had both died of cancer.

Homeopathic therapy was begun after considering all these factors and the individual symptomatology. The immunosuppressive drugs were stopped from the first day of homeopathic consultation, and the steroidal drugs were tapered slowly and completely stopped within one and a half months after starting homeopathic therapy.

## Outcome and follow-up

The patient steadily improved in terms of general condition (energy, appetite, weight and so forth) as well as blood reports (Table 2). As a result, she was able to increase the gap between consecutive dialysis sessions slowly, and eventually stop it in August 2016. Over the 28-month follow-up period, the patient was in a generally well-preserved condition with steady improvement in renal function.

**Table 2:** Evolution under homeopathic therapy

Date	Lab reports	Symptoms	Remedy	Changes in conventional interventions
**16/07/2015**	Serum creatinine: 5.2 mg/dl	Severe weakness Lost appetite Black discoloration of nails and skin since starting conventional treatment Face edema Weight loss in one year	Carcinosinum 30C, raised to 32C after one week and 33C after the next 10 days	Dialysis reduced to once a week; Withdrawal of immunosuppressive drugs
**27/08/2015**	Serum creatinine: 6 mg/dl (initial response to medicine brought about encouraging results, so the dialysis was postponed a little resulting in the temporary rise) Hb: 11.3 g%	Much more energy Black discoloration of nails and skin reduced considerably Increased urine output	Stop carcinosinum and wait	Steroids slowly tapered
**30/09/2015**	Serum creatinine: 5.14 mg/dl Blood pressure: 110/70mm Hg	Gained 2 kg Appetite improved	Nil	Withdrawal of antihypertensives
**25/11/2015**	Serum creatinine: 6.5 mg/dl	Generally very well	Nil	Is on dialysis once in 10 days, advised to continue the same
**20/01/2016**	Serum creatinine: 4.3 mg/dl	Gained 3.5 kg Improved energy and appetite Eruptions appeared on the left elbow (had them in the past)	Nil	Dialysis reduced to once a fortnight
**17/02/2016**	Serum creatinine: 5.5 mg/dl	Generally in good condition	Nil	Dialysis reduced to once a fortnight
**17/03/2016**	Serum creatinine: 5.6 mg/dl	Emotionally stressed after her mother died Developed acute lower respiratory tract infection - rattling cough, dyspnea, low appetite and energy	Ammoniacum gummi 30C	Nil
**13/04/2016**	Serum creatinine: 4.7 mg/dl	Generally very well	Nil	Nil
**04/05/2016**	Serum creatinine: 5.6 mg/dl Blood urea: 95.5 mg/dl	Low appetite for 15 days with nausea Weight reduced 1 kg Leg cramps Disturbed sleep Occasional loose stools	Sulphur 200C	Nil
**01/06/2016**	Serum creatinine: 4.9 mg/dl	Nausea absence Increased appetite More energy No loose stools No leg cramps Better sleep	Nil	Nil
**29/06/2016**	Serum creatinine: 4.7 mg/dl	Generally very well Increased skin eruptions on the elbow Dry skin, itching eczema on the right hand and knuckles	Nil	Increased gap between dialysis
**11/08/2016** **(one year after beginning homeopathy)**	Serum creatinine: 4.5 mg/dl RBC: 3.72X10^6^/cmm Hb: 11.6 g%	Weight increased by 2 kg Improved energy and appetite Eruptions increasing	Nil	Nil
**29/09/2016**	Serum creatinine: 3.7 mg/dl	Had fever for 2 days Improved energy Feels like before the onset of the disease Skin eruptions still increasing on the knees	Nil	Last dialysis on 24/08/2016 - AV fistula closed by accident and the patient did not return voluntarily for dialysis Took two paracetamol tablets in between for a mild fever
**01/12/2016**	Serum creatinine: 3.4 mg/dl Hb: 11.2 G% RBC: 3.56X10^6^/cmm SPO2: 97% Urine albumin ++	Nephrology assessment: Edema - nil Dyspnea - nil Appetite - good Urine - normal CVS/CNS - normal Blood pressure: 130/80 mm Hg	Nil	No dialysis for 3 months
**16/05/2017**	Serum creatinine: 3 mg/dl Hb: 10.9 g% Urine albumin: traces	Generally very well Skin eruptions are reducing	Nil	Nil
**03/11/2017**	Serum creatinine: 2.7 mg/dl	Reduced skin eruptions	Nil	Nil No dialysis since 14.5 months

The latest serum creatinine is 2.6 mg/dl, hemoglobin is 11.9g%, and the BVAS is estimated to be 4 and GFR is 19 mL/min/1.73 m^2^. The patient continues homeopathic therapy.

## Discussion

Renal dialysis is a considerably stressful procedure for patients, and it can be economically draining as well. It also carries the risk of various infections and iatrogenic complications [12, 13]. In severely affected RPGN, there is little else that can help alleviate the disease (except renal transplant), and the patient needs to undergo this cumbersome procedure regularly [6]. In the case of this 60-year-old patient, renal dialysis was altogether dispensed with, and the renal function was also restored to a certain extent in 28 months of follow-up.

The homeopathic perspective of a disease is holistic. It considers the whole process of inflammatory changes that have occurred from birth to the given point in time. ‘The Continuum of a Unified Theory of Diseases’ propounds the idea that all the pathological processes in a person from the time of birth to death form a continuum and are not incidental [14]. Initially, when the immune system is in a good state, it successfully mounts acute inflammation and drives away the pathogenic stimulus. When continuously thwarted through anti-inflammatory drugs or other substances that hinder inflammation, it loses the ability to react through strong inflammation and enters a state of chronic low-grade inflammation, eventually turning on the chronic disease that one is predisposed to. At such a stage, homeopathic therapy has been shown to reverse this situation, and as the chronic problem becomes simplified, the person begins to have acute diseases again [15–17]. Also, if any superficial inflammatory disease was suppressed through medication, at this point it returns, and such a return is a further confirmation of the immune system reverting to its previous state.

In this case, the patient had an episode of respiratory infection as the renal function started to improve, which was also treated by homeopathy. Further, she developed skin eruptions again, similar to what she had many years ago and was aggressively treated then. This time, the condition responded very well to the ongoing homeopathic treatment, and the eruptions have become less aggressive.

This rule of return of old suppressed conditions and onset of acute diseases as the chronic one becomes better is used as a guide by the homeopathic therapist to decide the direction the case is taking and to understand the prognosis in a given case [18, 19]. As it happens, in this case, the outcome was very good.

**Figure 1:**
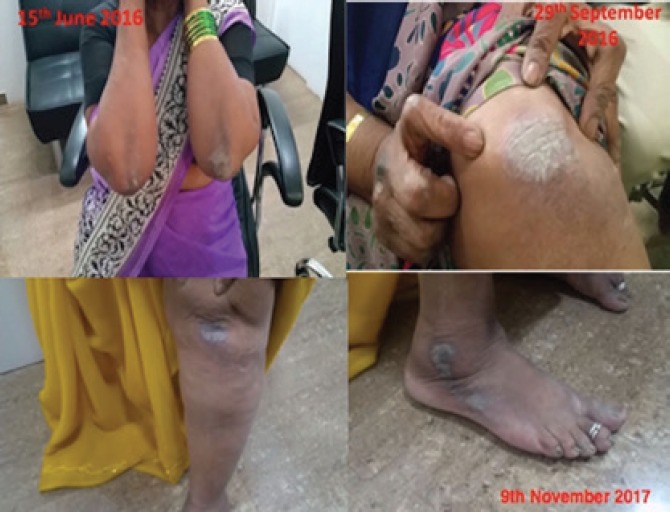
Onset and progress of the skin lesions during homeopathic therapy

The effect of homeopathic therapy is evident also in terms of pathology as is evidenced by the improvement of GFR from 8 mL/min/1.73 m^2^ to 19 mL/min/1.73 m^2^ and BVAS from 15 to 4.

The immunosuppressive drugs were stopped early on during homeopathic therapy resulting in elevated serum creatinine indicating the effect of immunosuppression withdrawal. However, this gradually responded to homeopathic therapy, and the patient was able to stop dialysis. The patient has remained free of drugs and dialysis for 20 months now, with steady improvement in renal function. This is reasonable grounds to plan more extensive controlled studies in the future.

Severe disorders and autoimmune conditions have been shown to respond to homeopathic therapy before now, and this case also indicates a possibility for this therapy [20–24]. There is a selection bias in this case as the patient opted for the treatment and the response was good. A controlled study will help establish the exact extent to which homeopathy may be of help and in this kind of cases.

## Conclusions

Even this rare case report of ANCA-negative RPGN showed a positive response to individualized homeopathic therapy while having poor prognosis conventionally. This suggests that RPGN may be amenable to individualized therapy in the early stages. Further, more extensive studies may establish the extent to which homeopathy may be useful in such conditions.

## Highlights

– ANCA-negative RPGN is challenging to treat even with immunosuppressive drugs and dialysis.
– Further proof for the questionable utility of plasmapheresis in the case of ANCA-negative RPGN.
– Classical individualized homeopathic therapy holds some promise in this pathology and needs to be investigated further.
– The phenomena of return of acute inflammatory states along with improvement in chronic inflammatory diseases are worth investigating to throw light on the immunological changes involved.

## Conflict of Interest

The authors confirm that there are no conflicts of interest.
